# Phenotypic and Functional Analyses Guiding Combination Immune Checkpoint Immunotherapeutic Strategies in HTLV-1 Infection

**DOI:** 10.3389/fimmu.2021.608890

**Published:** 2021-03-09

**Authors:** Danielle M. Clements, Brenndan Crumley, Glen M. Chew, Elijah Davis, Roberta Bruhn, Edward L. Murphy, Lishomwa C. Ndhlovu, Pooja Jain

**Affiliations:** ^1^Department of Tropical Medicine, John A. Burns School of Medicine, University of Hawaii, Honolulu, HI, United States; ^2^Department of Microbiology and Immunology, Drexel University College of Medicine, Philadelphia, PA, United States; ^3^Department of Medicine and Department of Laboratory Medicine, University of California at San Francisco, San Francisco, CA, United States; ^4^Vitalant Research Institute, San Francisco, CA, United States

**Keywords:** HTLV-1, HAM/TSP, PD-1, TIGIT, TIM-3, LAG-3, immune checkpoints, immunotherapy

## Abstract

Human T-cell lymphotropic virus type 1 (HTLV-1)-associated myelopathy/tropical spastic paraparesis (HAM/TSP) develops in 1–5% of HTLV-1-infected individuals. Previous studies by us and others have shown that the expression of negative immune checkpoint receptors (NCRs) is significantly increased on CD8 T cells in various chronic viral infections and are associated with poor anti-viral immunity. We have previously identified the differential expression of NCRs on CD8 T cells in blood from patients with HAM/TSP and in central nervous system (CNS) tissues of HTLV-1 infected humanized mice and defined the association with neurological complications. In this study, we determined the co-expression patterns of several key NCRs (PD-1, TIGIT, TIM-3, and LAG-3) and their cognate ligands in HTLV-1 infection and assessed how combination strategies targeting these pathways would impact HTLV-1-specific CD8 T-cell effector functions as an approach to reduce CNS disease outcomes. We found that global CD8 T cells from HAM/TSP patients co-express multiple NCRs at significantly higher frequencies than asymptomatic carriers (AC). Moreover, NCR ligands (PVR and PD-LI) on both plasmacytoid and myeloid dendritic cells were also expressed at higher frequencies in HAM/TSP compared to AC. In both AC and HAM/TSP subjects, combination dual PD-L1/TIGIT or triple PD-L1/TIGIT/TIM-3 blockade with monoclonal antibodies resulted in increases in intracellular cytokine expression in CD8 T cells after virus stimulation, particularly CD107a, a marker of degranulation, and TNF-α, a key cytokine that can directly inhibit viral replication. Interestingly, almost all blockade combinations resulted in reduced IL-2+ HTLV-1-specific CD8 T cell frequencies in HAM/TSP subjects, but not in AC. These results define a novel combinatorial NCR immunotherapeutic blockade strategy to reduce HAM/TSP disease burden.

## Introduction

Despite nearly 40 years since the discovery of human T-cell lymphotropic virus type 1 (HTLV-1), there is no vaccine or effective treatment for HTLV-1-associated myelopathy/tropical spastic paraparesis (HAM/TSP), a debilitating and progressive neurological disorder that manifests is 1–5% of infected individuals. This is, in part, due to a limited understanding of the interplay between viral and host factors that contribute to HTLV-1 disease pathogenesis. T-cell activation and effective downstream responses rely on two key events: recognition of antigen by the T-cell receptor and a second co-stimulatory signal. Immune checkpoint receptors on T cells modulate the magnitude and duration of activation by providing further stimulatory or suppressive signals (1). Suppressive signaling via negative immune checkpoint receptors (NCR) inhibits T cell responses, a means of controlling aberrant inflammation and potential damage to healthy tissues (2). Many diseases, especially cancers and chronic infections, hijack this system of immune checkpoints to dampen immune responses and avoid detection (2, 3). In the past decade, NCRs have been implicated as key contributors in several disease processes that culminate in T-cell “exhaustion,” where T cells are rendered dysfunctional and exhibit poor recall responses (3–6).

Cytotoxic T cells (CTLs) are an essential player in anti-viral immunity. The effectiveness of CD8 T cell responses against HTLV-1 infected cells could be the difference between asymptomatic infection and disease. The expression of NCRs, as well as their ligands, and the role they play in regulating CD8 T cell function in HTLV-1 is therefore important for understanding mechanisms that may be driving disease pathogenesis. The degree to which these surface receptors are expressed, how they are regulated, and whether or not they can be targeted to reverse T cell inhibition requires further exploration so that more effective therapies can be developed to reduce disease burden.

This study assessed, expression of programmed cell death protein 1 (PD-1), T cell immunoreceptor with Ig and ITIM domain (TIGIT), T-cell immunoglobulin and mucin-containing domain 3 (TIM-3) and lymphocyte-activation gene 3 (LAG-3) on T cells, along with ligands for PD-1 and TIGIT on dendritic cells. Several of these NCRs have been shown to be expressed on T cells during HTLV-1 infection (7). PD-1 and TIGIT are elevated on HTLV-1-specific CD8 T cells in adult T-cell leukemia (ATL), another disease that can result from HTLV-1 infection (8), as well as HAM/TSP patients (9, 10). While expression of PD-1 and TIGIT are known to be increased, the expression of other NCRs like TIM-3, B and T lymphocyte attenuator (BTLA) and leukocyte-associated immunoglobulin-like receptor 1 (LAIR-1) are reduced, indicating a selective and variable NCR profile in HTLV-1 infection (11, 12). PD-L1, the ligand for PD-1, is also expressed at elevated levels in ATL. Recently, a study using a monoclonal antibody targeting PD-1 resulted in rapid ATL progression in HTLV-1-infected individuals who received the antibody treatment (13). PD-1 normally functions as a tumor suppressor in T-cell lymphomas (14). However, the tumor-suppressive effects of PD-1 are seemingly hindered in HTLV-1-infected cells, emphasizing the need to carefully assess the hierarchy of NCRs, and the co-expression of PD-1 with other NCRs in HTLV-1. Furthermore, reports of NCR expression patterns sometimes conflict in the literature, making it difficult to determine which immune checkpoint pathways are important in HTLV-1 pathogenesis and rational targets for immunotherapeutic strategies.

Given the expanding breadth and complexity of NCRs it remains unclear which combination of inhibitory receptors/ligands are most important in T cell regulation during HTLV-1 infection and whether individual or combination blockade of NCR-ligand interactions is sufficient for restoring CTL function (15–18). The promising results seen with immune checkpoint blockade against a growing list of cancers (19, 20) further supports the use of checkpoint blockade as a potential therapy for HAM/TSP. Thus, we investigated the expression profiles of several NCRs in both asymptomatic and symptomatic individuals infected with HTLV-1 and assessed combination blockade strategies that may lead to novel immunotherapeutic strategies for treating HTLV-1 neurological disease.

## Methods

### Ethics Statement

All study participants gave written informed consent to participate in the University of California San Francisco (UCSF) HTLV-1 Outcomes Study (HOST) and approval was obtained from the University of Hawaii Human Studies Program to conduct analyses on banked specimens obtained from the study.

### Study Cohort

A subset of 38 participants from a larger, prospective, multi-center cohort of individuals with HTLV infection were used in this study (Table 1). Briefly, the UCSF HOST cohort consists of individuals who were found to be HTLV seropositive at the time of attempted blood donation at 5 major US blood centers between 1990 and 1992, as well as seronegative donors enrolled at the same centers. Details of cohort enrollment have been previously published (21). HTLV-1+ participants (*n* = 26) all had detectable HTLV-1 infection confirmed by Western Blot and PCR (for HTLV-1 typing). HTLV-1 seronegative controls (SC) were matched ~2:1 to HTLV-1+ participants based on age, sex, race or ethnicity, and blood center. The HTLV-1+ group consisted of asymptomatic carriers (AC) and individuals who developed HAM/TSP (HAM/TSP).

**Table 1:** Patient characteristics.

	**HTLV-1 negative**		**HTLV-1 positive**			
	**SCs** **(*n* = 12)**	**AC** **(*n* = 20)**	**HAM/TSP** **(*n* = 6)**	**Total** **(*n* = 26)**	**AC vs. HAM/TSP** ***p*-values**	**HTLV-1+ vs. HTLV-1+** ***p* values**
Gender distribution % (*n*)					>0.999	>0.999
Male	17 (2)	15 (3)	17 (1)	15 (4)	–	–
Female	83 (10)	85 (17)	83 (5)	85 (22)	–	–
Age (years) mean, SD	46.2 ± 8.3	46.5 ± 7	45.2 ± 8.8	46.2 ± 7.3	0.639	0.930
Race % (*n*)					0.9172	0.9191
Asian	16.6 (2)	10 (2)	16.6 (1)	11.5 (3)	–	–
Black	33.3 (4)	35 (7)	33.3 (2)	34.6 (9)	–	–
Hispanic	16.6 (2)	10 (2)	16.6 (1)	11.5 (3)	–	–
White	33.3 (4)	45 (9)	33.3 (2)	42.3 (11)	–	–
Time since diagnosis (years) mean, SD	–	–	4.5 ± 2.7	–	–	–
Proviral load (copies/100 cells) median (min, max)	0 (0, 0)	27 (0, 1,740)	610 (161, 861)	72.5 (0, 1,740)	0.0112	–

*SC, Seronegative Control; AC, Asymptomatic Carrier*.

### Quantification of HTLV-1 Proviral Load

HTLV-1 proviral loads were measured from cryopreserved peripheral blood mononuclear cells (PBMCs) from all HTLV-1+ specimens and three seronegative controls as described by Furtado et al. (22). Briefly, cellular DNA was isolated from PBMCs by column extraction using the QIAamp DNA Mini Kit (Qiagen, Ventura, CA). HTLV-1 proviral load was quantified via real-time PCR on an ABI Prism 7300 Sequence Detector System (Applied Biosystems In., Foster City, CA). The 186-bp fragment of the *pol* gene was amplified using SK110 forward (5′-CCCTACAATCCAACCAGCTCAG-3′) and SK111 reverse (5′- GTGGTGAAGCT GCCATCGGGTTTT-3′) primers. To calculate the number of HTLV-1 copies per cell, the albumin gene was quantified in parallel separate reactions using ALB-S forward (5′-GCTGTCATCTCTTGTGGGCTGT-3′) and ALB-AS reverse (5′-AAACTCATGGGAGCT GCTGGTT-3′) primers. Approximately 240 ng of DNA were used in each reaction with 1X SYBR Green PCR Master Mix (Applied Biosystems) and 200 nM of each primer. Cycling conditions were 2 min at 50°C and 10 min at 95°C followed by 40 cycles of 15 s at 95°C and 1 min at 65°C. Specimens were assayed in duplicate reaction wells and copy number was determined by extrapolation against a 6-point standard curve (1–100,000 copies) generated from serial DNA dilution from MT2 cells and normalized to three copies of HTLV-1 *pol* gene and two copies of albumin gene per MT2 cell. Values for HTLV-1 proviral load are reported as (pol average copy number)/(albumin average copy number/2) × 10^2^ cells.

### Immunophenotyping and Flow Cytometric Analysis

Cryopreserved PBMCs were rapidly thawed in complete RPMI (cRPMI, Hyclone, Logan, UT) [RPMI 1640 medium supplemented with 10% heat-inactivated fetal bovine serum (FBS) (Hyclone), 1% penicillin-streptomycin (Hyclone), 10 mM HEPES (Hyclone) and 2 mM L-glutamine (Hyclone)] followed by two washes in cRPMI. Cells were then stained for viability using yellow or aqua amine reactive dyes (YARD/AARD; Invitrogen, Carlsbad, CA) in 1X phosphate buffered saline (PBS, Hyclone). Fluorochrome-conjugated anti-human monoclonal antibodies (mAbs) were then used to stain cells for various surface markers in 1X PBS/2% FBS. The following mAbs were used in various panels: from BD Biosciences (San Jose, California) Brilliant Violet 510-conjugated anti-CD4 (OKT4), Flourescein isothiocyanate (FITC)-conjugated anti-CD8 (HIT8a), Phycoerythrin (PE)-conjugated anti-CD151 (14A2.H1), PE-Cy7-conjugated anti-CD19 (SJ25C1), PE-Cy7-conjugated anti-CD20 (2H7), Qdot 605-conjugated anti-CD8, APC-conjugated anti-CD57 (HCD57), V450-conjugated anti-CD45RA (HI100), PerCP-Cy5.5-conjugated anti-CD3 (SK7), PE-conjugated anti-PVR (SKII.4), PE-Cy7-conjugated anti-CD7 (6B7), APC-conjugated anti-HLA-DR (G46-6), FITC-conjugated anti-Ki67 (35/Ki67); from BioLegend (San Diego, CA), Brilliant Violet 711-conjugate anti-CD3 (OKT3), Brilliant Violet 605-conjugated anti-CD14 (M5E2), PerCP-eFluor 710-conjugated anti-TIGIT (MBSA43), APC-Cy7-conjugated anti-PD-1, Alexa Fluor 700-conjugated anti-CD4 (RPA-T4), Alexa Fluor 647-conjugated anti-CCR7 (G043H7), Brilliant Violet 421-conjugated anti-PD-L1 (29E.2A3), Brilliant Violet 510-conjugated anti-CD11b (ICRF44), Brilliant Violet 605-conjugated anti-CD14 (M5E2), Brilliant Violet 711-conjugated anti-CD16 (3G8), FITC-conjugated anti-CD123 (7G3); from Invitrogen/eBioscience (San Diego, CA), Super Bright 645-conjugated anti-LAG-3 (3DS223H), PE-Cy7-conjugated anti-CD28 (CD28.2), FITC-conjugated anti-LAG-3 (3DS223H), Alexa Fluor 700-conjugated CD11c (3.9); from R&D Systems (Minneapolis, MN), PE-conjugated anti-TIM-3 (344823); from Beckman Coulter (Fullerton, CA), ECD-conjugated anti-CD3 (UCHT1). CCR7 staining included an incubation at 37°C for 10 min prior to surface staining. For panels that included Ki67, cells were fixed and permeabilized using 1X Lyse Buffer (BD Biosciences) and 1X BD FACS Permeabilizing Solution 2 (BD Biosciences), then stained with FITC-conjugated anti-Ki67 (35/Ki-67). Cells were washed twice after staining with 1X PBS/2% FBS and fixed in 1% paraformaldehyde (PFA, Electron Microscopy Sciences, Hatfield Pennsylvania) before acquiring on a custom four laser LSRFortessa flow cytometer (BD Biosciences) using FacsDiva software (BD Bioscience). Between 50,000 and 500,000 events (based on a live lymphocyte gate) were collected for each sample. UltraComp eBeads (Thermo Fisher Scientific, Carlsbad, CA) were stained with each fluorochrome-conjugated antibody for software-based compensation, and Flowjo Version 10.5.3 (Treestar, Ashland, OR) was used for flow analysis. Fluorescence minus one (FMO) samples were used to approximate gate placement (Supplementary Figure 1).

### Pentamer Staining

Cryopreserved PBMCs were rapidly thawed as described above and stained with APC labeled HTLV-1 Tax A^*^02:01 LLFGYPVYV Pentamer from Proimmune Ltd. (Oxford, UK). After incubation for 10 min, cells were washed with PBS/ 0.1% BSA (MilliporeSigma, St. Louis, MO) and stained with a surface antibody cocktail against CD3, CD4, CD8, CD14, CD19, CD20, PD-1, TIGIT, TIM-3, and LAG-3. Cells were washed twice with 1X PBS/2% FBS before acquisition on a three-laser BD FACS ARIA (BD Biosciences). FlowJo v10.3 (BD, Ashland, OR) was used for flow analysis. FMO samples were used to approximate positive gates.

### Anti-PD-L1, Anti-TIGIT, Anti-TIM-3, and Anti-LAG-3 Monoclonal Antibodies

Anti-PD-L1 and TIGIT monoclonal blocking antibodies were gifted from Bristol-Myers Squibb (BMS) and are previously described in (23). Anti-TIM-3 and LAG-3 mAb were obtained through an LCN-initiated industry collaboration.

### HTLV-1 Virion Generation From MT-2 Cells

MT-2 cells obtained through the NIH AIDS Reagent Program were used to generate HTLV-1 virus. MT-2 cells were grown in RPMI medium supplemented with 10% FBS (Hyclone) and supernatant was filtered through a 0.45 um polyethersulfone (PES) low protein-binding filter (Millipore) before being concentrated using Retro-X Concentrator (Takara Bio, Japan). Briefly, virus-containing supernatant was incubated overnight at 4°C at a ratio of 3 volumes of clarified MT-2 supernatant to 1 volume of Retro-X Concentrator followed by centrifugation at 1,500 g for 45 min at 4°C. The virus-containing pellet was resuspended in a volume of 1X PBS equivalent to the 1/100th the original volume of supernatant. An HTLV-1 p19 antigen ELISA (ZeptoMetrix Corp., Buffalo, NY) was used to determine the final concentration of virus. Patient samples were stimulated with concentrated virus at a final concentration of 100 ng/mL.

### *In vitro* NCR Blockade Assay

For *in vitro* blockade assays, cryopreserved PBMCs from AC (*n* = 17) and HAM/TSP patients (*n* = 5) were rapidly thawed and cultured in 96-well round-bottom plates at ~5 × 10^5^ cells per well in RPMI. Brefeldin A and monensin (MilliporeSigma) were added to each well at 5 ug/mL along with MT-2 cell-derived HTLV-1 whole virus or anti-CD3/anti-CD28 Dynabeads (Life Tech, San Diego). Isotype controls, anti-PD-L1, anti-TIGIT, anti-TIM-3, and anti-LAG-3 mAbs were added separately or in combination to appropriate wells. After overnight incubation, cells were washed and stained for viability then incubated with anti-CD8 antibody. Cells were fixed using 1X Lyse buffer and permeabilized using 1X BD FACS permeabilizing solution before being stained for intracellular markers including CD3, IFN-γ, CD107a, TNF-α, and IL-2. Cells were washed twice with PBS/2% FBS before fixation with 1% PFA. Cells were acquired on a custom four laser LSRFortessa flow cytometer (BD). UltraComp eBeads (Thermo Scientific, Carlsbad,) were individually stained with each fluorochrome-conjugated antibody for software-based compensation. Data was analyzed using FlowJo v10.3. The gating strategy used can be found in Supplementary Figure 1.

### Statistical Analysis

One-way ANOVAs using Kruskal-Wallis and Dunn's multiple comparisons test were used for comparison of groups, Wilcoxon matched-pairs signed rank test for paired samples, and Mann-Whitney test for unpaired samples. Spearman's rho test was used for correlation analyses. Means are shown with standard deviation and central tendency is expressed as median and interquartile range. Statistical analyses were conducted with GraphPad Prism Version 8.0.1 (GraphPad Software, San Diego, CA), using *p* = 0.05 as the threshold for statistical significance.

## Results

### Single and Co-Expressed Negative Immune Checkpoint Receptors Are Upregulated on CD4 and CD8 T Cells During HTLV-1 Infection, but Not on Virus-Specific CD8 T Cells

**Figure 1:**
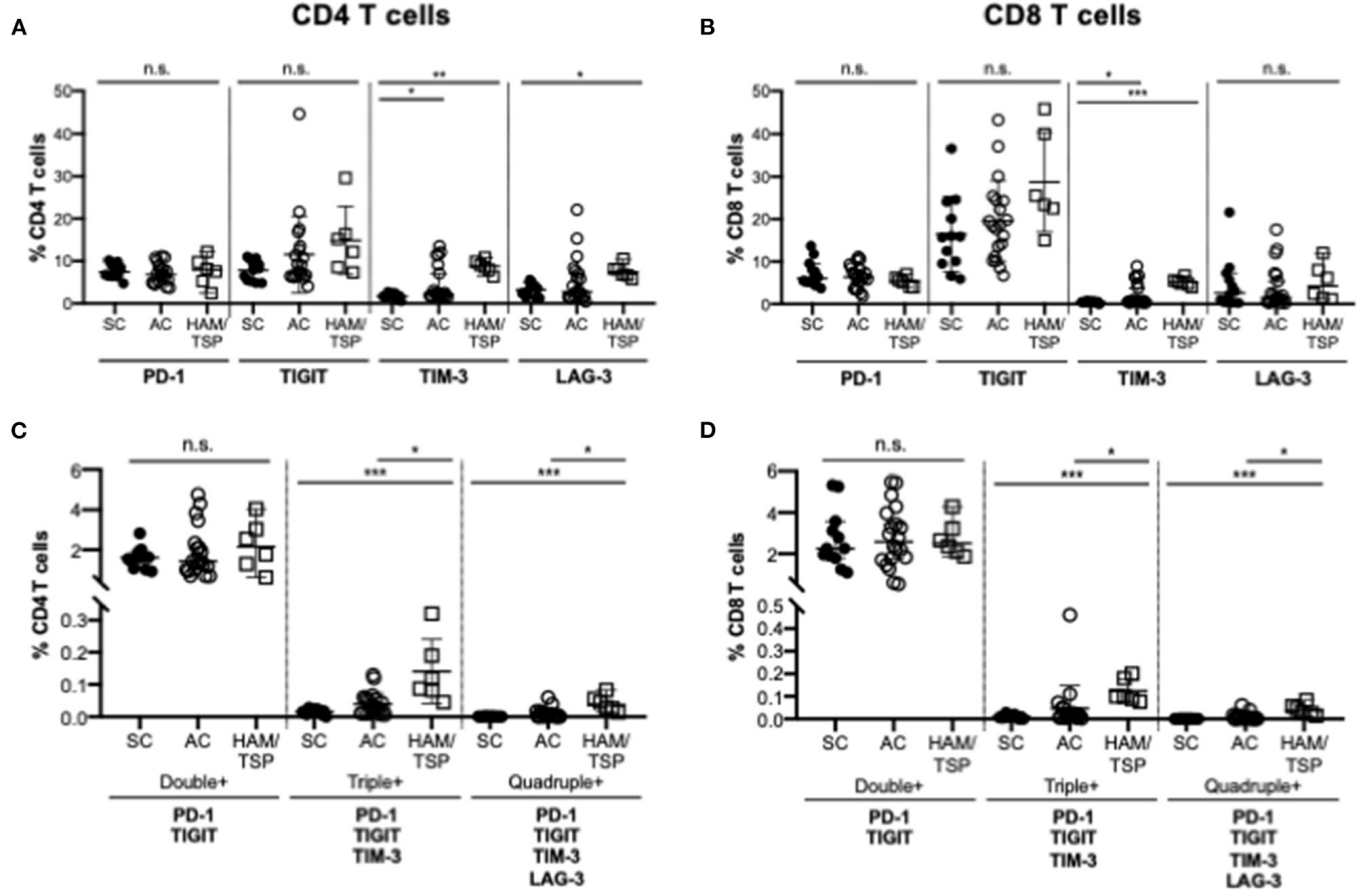
NCR expression on T cells in HTLV-1 infection. Cryopreserved PBMCs from HTLV-1-infected asymptomatic carriers (AC, *n* = 20), HTLV-1-infected individuals diagnosed with HAM/TSP (HAM/TSP, *n* = 6) and seronegative controls (SC, *n* = 12) were thawed and immunophenotyped for NCR expression on CD4 and CD8 T cells. Frequency of single expression of PD-1, TIGIT, TIM-3 and Lag-3 on CD4 T cells **(A)**, CD8 T cells **(B)** are shown in the upper panels. Lower panels show frequency of multiple negative checkpoint receptor expression on CD4 T cells **(C)** and CD8 T cells **(D)**. Double NCR expression includes PD-1 and TIGIT; triple NCR expression includes PD-1, TIGIT, TIM-3; quadruple NCR expression includes PD-1, TIGIT, TIM-3, LAG-3. Multiple NCR expression was determined using Boolean gating. Significance was calculated using Kruskal-Wallis multiple comparisons test (*p < 0.05; **p < 0.005; ***p < 0.0005).

To evaluate the relative expression of single and multiple co-expressed NCRs on CD4 and CD8 T cells during HTLV-1 infection, cryopreserved PBMCs from HTLV-1-infected asymptomatic carriers (AC, *n* = 20), HTLV-1-infected individuals diagnosed with HAM/TSP (HAM/TSP, *n* = 6) and seronegative controls (SC, *n* = 12) were assessed for expression of PD-1, TIGIT, TIM-3, and LAG-3 (Table 1, Figure 1; Supplementary Figure 1). We observed significantly increased global single TIM-3^+^ and LAG-3^+^ CD4 T cells and TIM-3^+^ CD8 T cells in HAM/TSP subjects when compared to SN controls (Figures 1A,B). PD-1 expression on CD4 and CD8 T cells also differed between groups. Although TIGIT expression trended higher between the HTLV-1 infected groups and SC, this was not significant. Among HTLV-1-infected individuals, there were no significant differences in single NCR expression on global CD4 and CD8 T-cell populations. However, we did observe that triple (PD-1^+^TIGIT^+^TIM-3^+^) and quadruple (PD-1^+^TIGIT^+^TIM-3^+^LAG-3^+^) co-expressing CD4 and CD8 T cells were significantly increased in HAM/TSP subjects when compared to both AC and SC groups (Figures 1C,D). No significant differences were observed for dual PD-1^+^TIGIT^+^ NCR co-expression on either CD4 or CD8 T cells.

**Figure 2:**
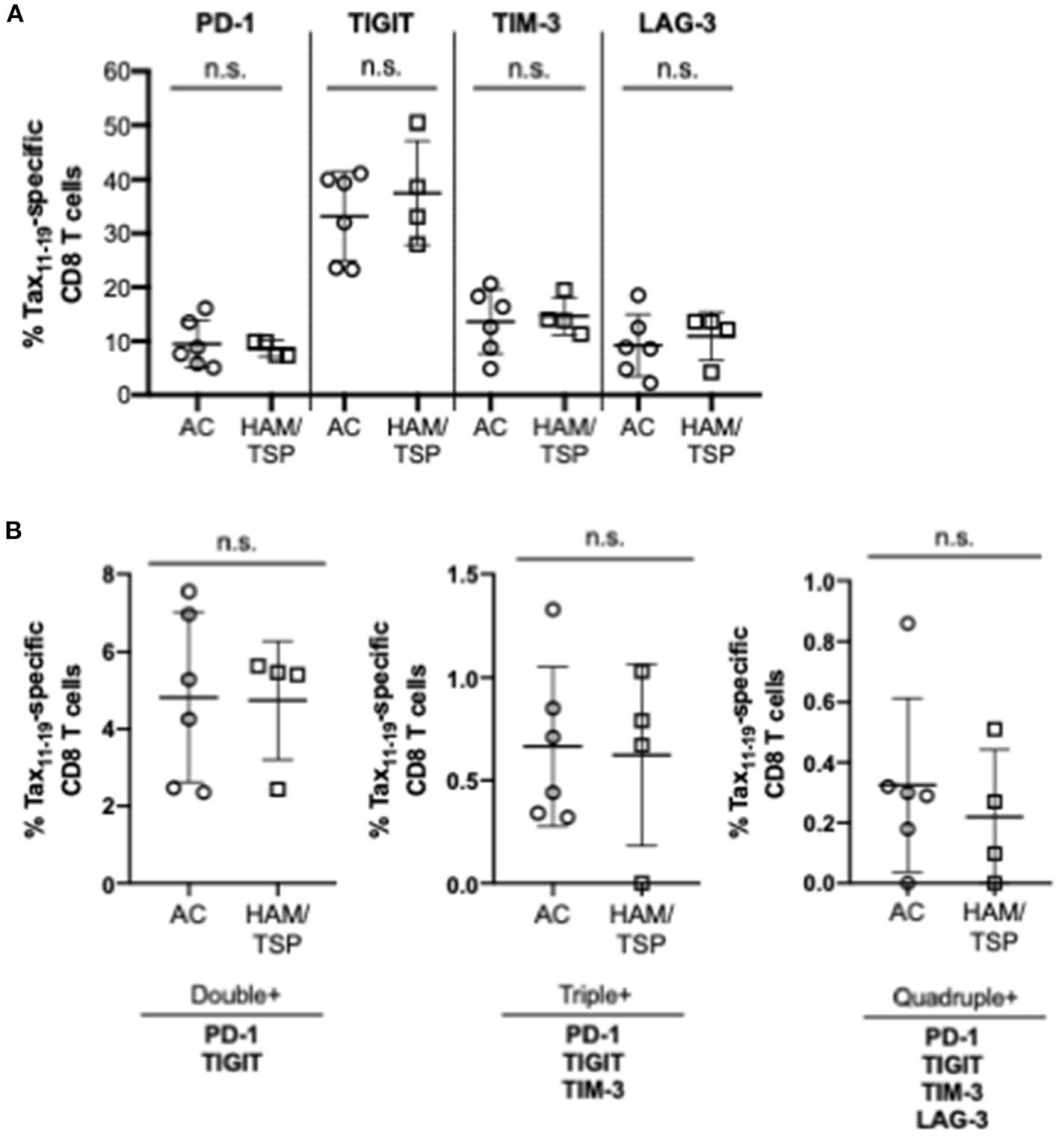
NCR Expression on HTLV-1 Tax-specific CD8 T cells. Cryopreserved PBMCs from all HTLV-1-infected asymptomatic carriers (AC, *n* = 6) and HTLV-1-infected individuals diagnosed with HAM/TSP (HAM/TSP, *n* = 4) were thawed and stained with HTLV-1 Tax A*02:01 LLFGYPVYV MHC Class I pentamer before being stained for surface expression of single **(A)** and multiple NCRs **(B)**. Significant differences between AC and HAM/TSP were assessed using Mann-Whitney test with *p*-value < 0.05 considered to be significant. No statistically significant differences were observed.

We next examined the expression of the NCR on HTLV-1 Tax_11−19_-specific CD8 T cells; this was limited to donors with HLA-type (HLA-A^*^02:01) specificity. Tax_11−19_-specific positive gates were set based on HTLV-1 negative controls (HTLV-1 and HIV+ samples) and needed to exceed a frequency threshold of 0.4% of total CD8 T cells (Figure 2; Supplementary Figure 2). We observed no difference in single or multiple NCR co-expression on HTLV-1 Tax_11−19_-specific CD8 T cells between the AC and HAM/TSP group (Figures 2A,B).

### T Cells Expressing Multiple NCRs Are More Prevalent in Individuals With HAM/TSP in Differentiated Memory T-Cell Subsets

While CD8 T-cell function can become compromised during chronic viral infection, HTLV-1 predominantly infects CD4 T cells, so it was important to consider NCR expression in both T-cell populations and in various differentiated T-cell subsets. First single NCR expression profiles were compared between groups (Supplementary Figures 3–6). PD-1 expression on CD4 and CD8 T cells was highest in the transitional memory (TM) T-cell subset, with significant differences observed only in the central memory (CM) subset of CD8 T cells between AC and HAM/TSP (Supplementary Figure 3). TIGIT expression was highest on TM CD4 T cells and significantly higher on naïve and TM CD4 T cells in HAM/TSP (Supplementary Figure 4). The frequency of TIGIT expression on CD8 T cells increased as cells became more differentiated, with the highest expression observed on terminally differentiated (TD) T cells. TIM-3 expression on both CD4 and CD8 T cells was significantly higher in HAM/TSP subjects compared to AC across all differentiated T-cell subsets, with highest expression seen on effector memory (EM) and TD subsets (Supplementary Figure 5). Interestingly, TIM-3 expression was significantly lower in AC than SC, especially on CD8 T cells, which partially agrees with TIM-3 expression patterns reported in literature (11, 12). LAG-3 expression was higher on naïve CD4 and CD8 T cells and in CM CD8 T cells in HAM/TSP and no significant differences were observed between groups in other memory T-cell subsets (Supplementary Figure 6). Together, these data suggest that single expression of NCRs on select memory T-cell subsets may be more advantageous for virus survival.

**Figure 3:**
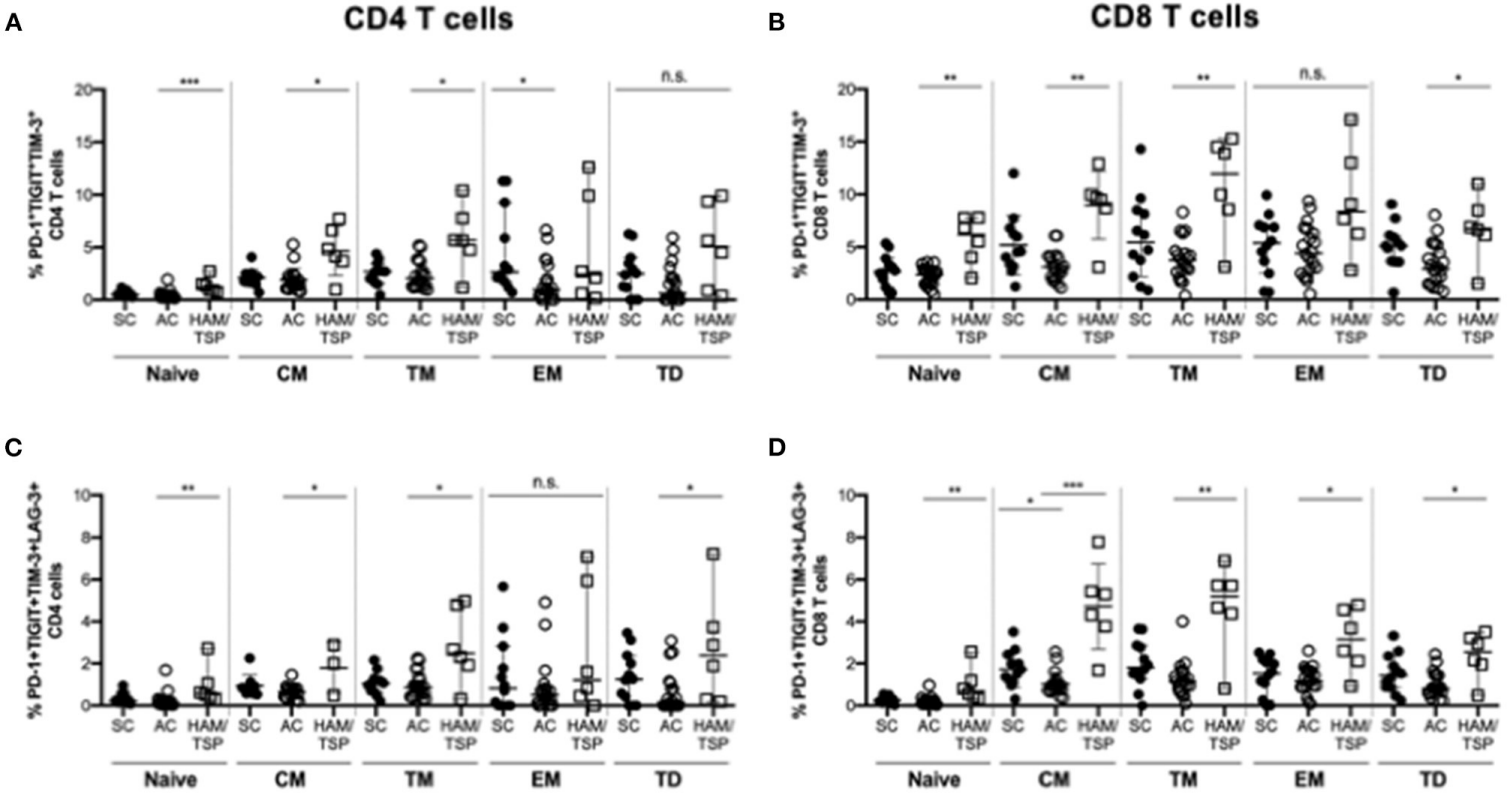
Multiple NCR expression on differentiated T-cell subsets. Cryopreserved PBMCs were surface stained for multiple NCRs co-expressed on various CD4 **(A,C)** and CD8 **(B,D)** T-cell subsets in SC, AC and individuals with HAM/TSP. Graphs show frequency CD4 **(A)** and CD8 **(B)** T-cell subsets simultaneously co-expressing PD-1, TIGIT, TIM-3 as well as CD4 **(C)** and CD8 **(D)** T-cell subsets quadruple positive for PD-1, TIGIT, TIM-3, LAG-3. T-cell subsets were defined as naïve (CD28+CD45RA+CCR7+), central memory (CM, CD28+CD45RA-CCR7+), transitional memory (TM, CD28+CD45RA-CCR7-), effector memory (EM CD28-CD45RA-CCR7-) and terminally differentiated (TD, CD28-CD45RA+CCR7-). Significant differences between groups were calculated using Kruskal-Wallis multiple comparisons test with *p*-values < 0.05 considered to be significant (*p < 0.05; **p < 0.005; ***p < 0.0005).

When evaluating multiple NCR expression in memory T-cell subsets, we observed that dual (PD-1^+^TIGIT^+^) TM CD4 T cells were significantly increased in HAM/TSP donors compared to the SC group (Supplementary Figure 7). Only dual (PD-1^+^TIGIT^+^) CM CD8 T cells were expanded in HAM/TSP donors compared to AC. Triple (PD-1^+^TIGIT^+^TIM-3^+^) and quadruple (PD-1^+^TIGIT^+^TIM-3^+^LAG-3^+^) NCR expression had significantly higher frequencies in the HAM/TSP group across almost all memory subsets on both CD4 and CD8 T cells (Figure 3). Triple NCR co-expression was found at higher frequencies on naïve, CM and TM, but not EM and TD CD4 T cells in HAM/TSP compared to AC. Similarly increased quadruple NCR expression was seen across all memory CD4 T-cell subsets in HAM/TSP group, except on EM CD4 T cells. Broadly, naïve, CM, TM and TD CD8 T cells showed higher frequencies of triple and quadruple NCR expression in HAM/TSP subjects, with quadruple NCR expression also significantly higher on EM CD8 T cells in HAM/TSP group compared to SC.

### TIGIT and PD-1/TIGIT Co-Expression on CD4 T Cells Correlates With HTLV-1 Proviral Load

**Figure 4:**
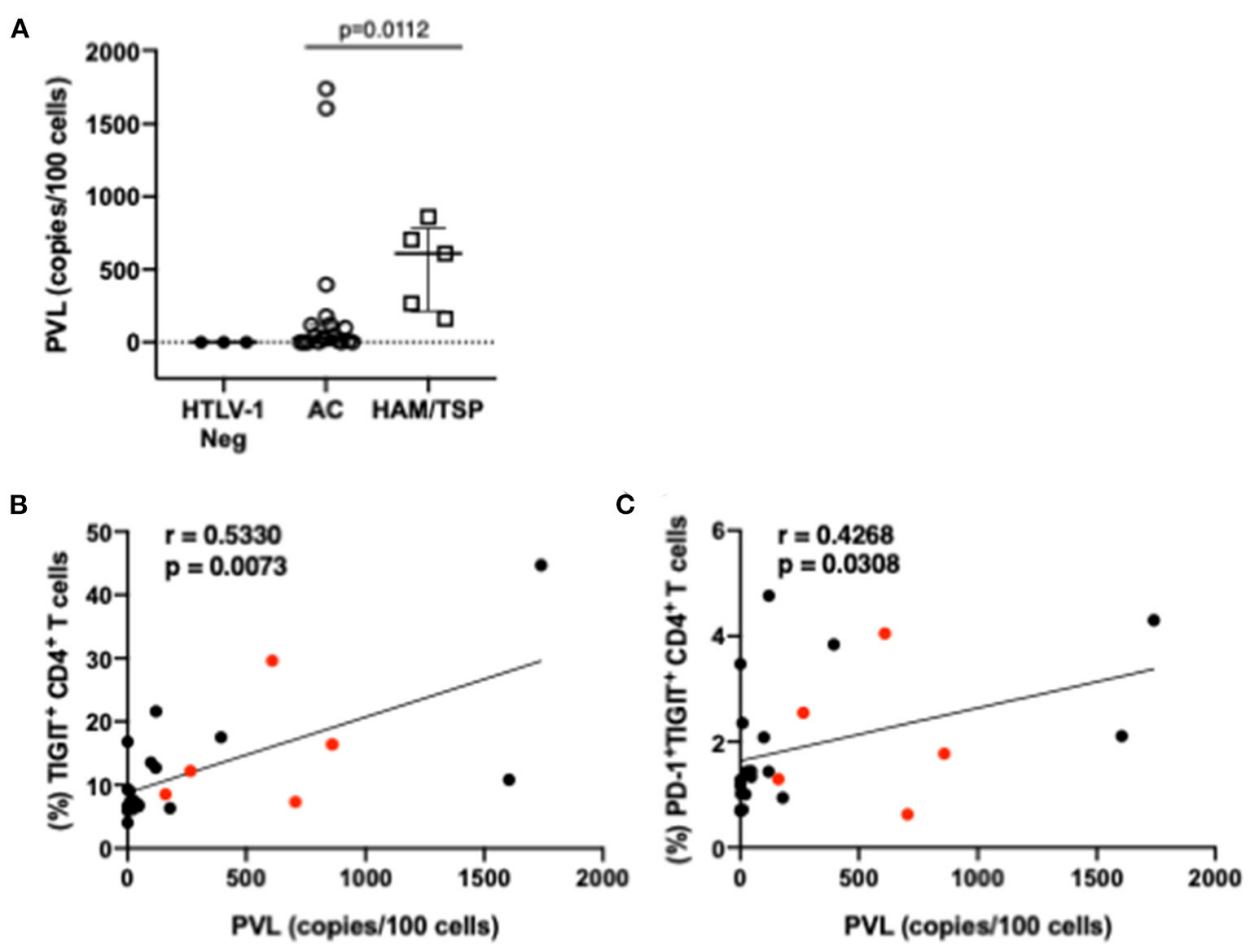
Proviral load and correlations with NCR expression. Proviral load was measured from whole PBMCs of HTLV-1-seropositive study participants (AC and HAM/TSP) as well as three HTLV-1-seronegative controls **(A)**. Real-time PCR amplification of a 185-bp fragment of the pol gene was conducted in parallel with albumin, which served as a reference gene. Proviral loads were calculated using a standard curve and reported as copies per 100 cells. Frequencies of NCR expressing T cells from all HTLV-1+ study participants were correlated with proviral load measurements to determine if any relationship between proviral load and NCR expression exists. Lower panels show correlations with significant *p*-values (<0.05): frequency of TIGIT+ CD4 T cells vs. proviral load **(B)** and frequency of dual PD-1+TIGIT+ CD4 T cells **(C)**. Red dots indicate individuals with HAM/TSP; black dots are AC. Spearman's rho tests were performed for correlations **(B,C)** and student *t*-test for difference in proviral loads amongst HTLV-1 positive study participants. *P*-values < 0.05 considered to be significant.

HTLV-1 proviral load has been shown to correlate with HTLV-1 disease progression. Accordingly, significantly higher proviral loads were observed in individuals with HAM/TSP compared to AC (Figure 4A) in our cohort. Two ACs had high viral loads and may identify individuals that may progress to HAM/TSP, although this could not be confirmed. Frequencies of single and multiple NCR-expressing T cells were correlated with proviral load measurements (Figures 4B,C). Higher proviral load was found to correlate with higher frequency of single TIGIT-expressing CD4 T cells as well as co-expression of PD-1 and TIGIT on CD4 T cells. We did not observe any other significant correlations between proviral load and other single or multiple co-expressed NCRs on CD8 T cells.

### NCR Ligand Expression on Myeloid and Plasmacytoid Dendritic Cells Is Increased in HAM/TSP Subjects

**Figure 5:**
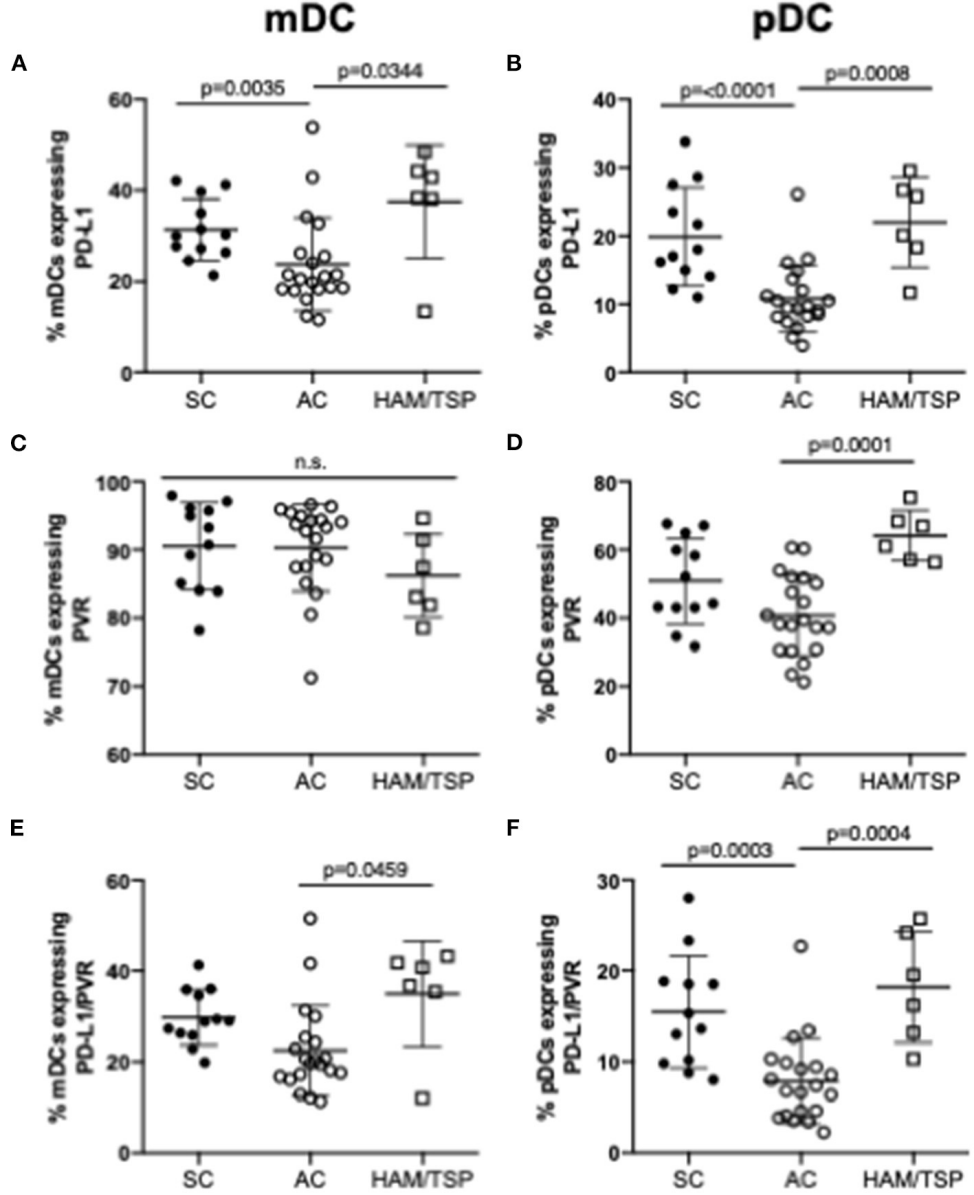
Expression of NCR ligands on dendritic cells in HTLV-1 infection. Cryopreserved PBMCs from SC, AC and HAM/TSP were immunophenotyped for assessment of NCR ligand expression on two dendritic cell (DC) lineages, myeloid DC (mDCs) **(A, C, E)** and plasmacytoid DC (pDCs) **(B, D, F)**. mDCs were defined as Lin- (CD3-CD14-CD7-CD19-CD20-) CD11c+CD123- cells; pDCs were defined as Lin- CD11c- CD123+ cells. PD-L1 (PD-1 ligand) **(A,B)** and PVR (TIGIT ligand) **(C,D)** single expression and co-expression **(E,F)** are shown. Mann-Whitney tests were used to determine statistical differences between groups. *P*-values < 0.05 considered to be significant.

PD-L1 and PVR, the respective ligands for PD-1 and TIGIT, are upregulated in cancer and chronic viral infection. The expression of these ligands on myeloid (mDCs) and plasmacytoid dendritic cells (pDCs) were assessed since availability of NCR ligands may also affect NCR-mediated T-cell dysfunction. mDCs and pDCs were immunophenotyped based on CD11c and CD123 expression: mDCs were defined as Lin- (CD3^−^CD14^−^CD7^−^CD19^−^CD20^−^) CD11c^+^CD123^−^ cells; pDCs as Lin- CD11c^−^ CD123^+^ cells. We observed increased PD-L1 expression on both mDCs and pDCs in HAM/TSP subjects, while PVR expression increased in HAM/TSP subjects on pDCs only when compared to AC (Figures 5A–D). Notably, there was a clear tendency, sometimes significantly so, toward lower expression of NCR ligands compared to SC, while NCR ligand expression in the HAM-TSP group was similar to the SC controls. Compared to AC, the co-expression of dual PD-L1 and PVR on both mDCs and pDCs was higher in the HAM/TSP group (Figures 5E,F). Finally, significantly decreased dual NCR ligand co-expression on both mDCs and pDCs was seen in AC when compared to SC group (Figure 5F).

### HAM/TSP Subjects Show Higher Levels of Anti-HTLV-1 CD8 T-Cell Cytokine Responses

**Figure 6:**
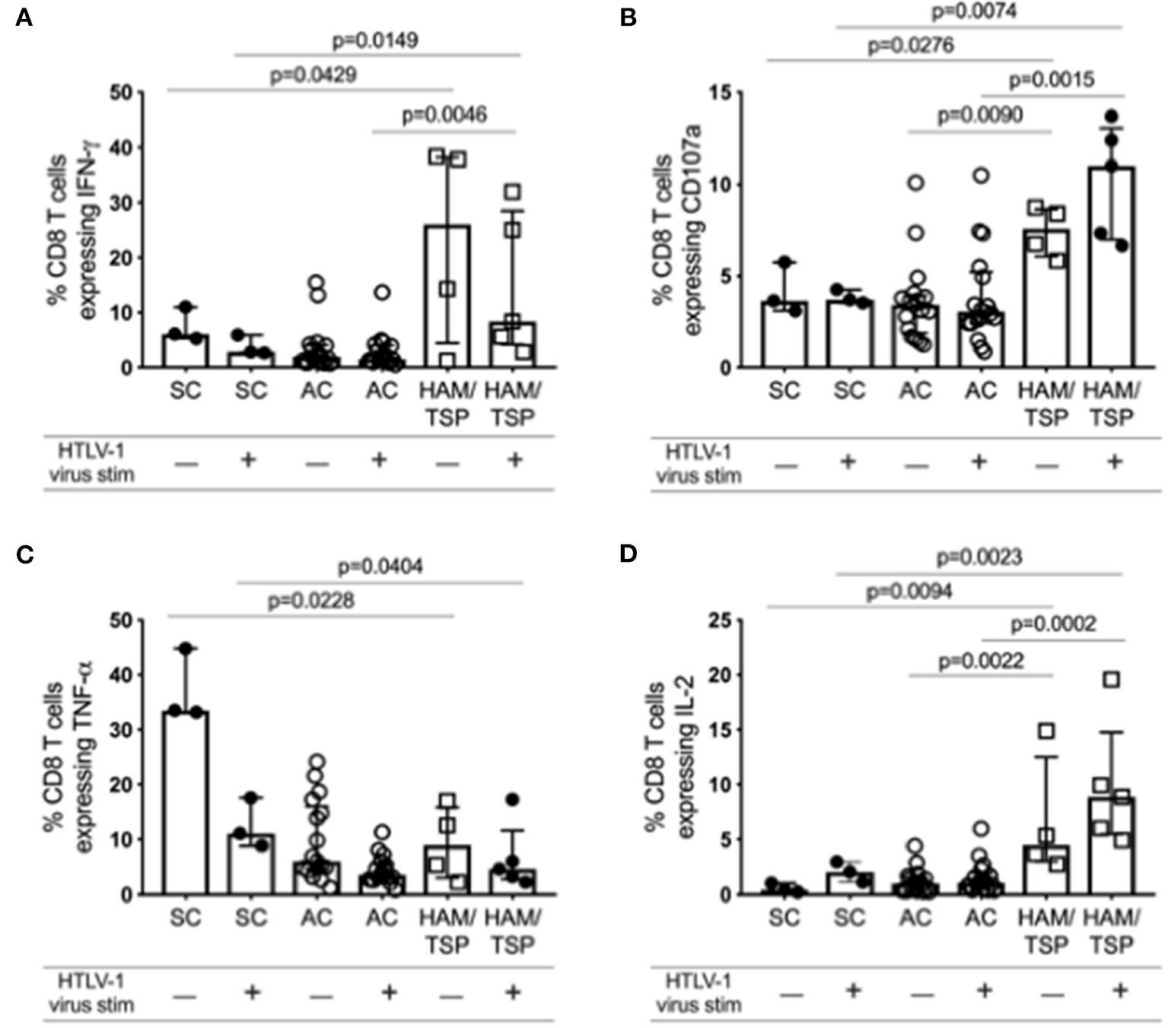
*Ex vivo* cytokine expression profile of CD8 T cells during HTLV-1 infection. Cryopreserved PBMCs from 17 AC, 5 HAM/TSP patients and 3 SC were immunophenotyped for various intracellular cytokine expression in CD8 T cells after stimulation with PBS (unstimulated) or with HTLV-1 derived from MT-2 cell culture supernatants for 16 h. Frequency of CD3+CD8+ T cells expressing IFN-γ **(A)**, CD107a **(B)**, TNF-α **(C)**, and IL-2 **(D)** are shown. Significant differences between groups were calculated using Dunn's multiple comparisons test with *p*-values < 0.05 considered to be significant.

Despite increased NCR expression on both CD4 and CD8 T cells in HAM/TSP subjects compared to SC and AC, indicating potential T-cell exhaustion, we observed markedly higher cytokine expression in CD8 T cells before and after stimulation in HAM/TSP subjects (Figure 6; Supplementary Figure 8). IFN-γ, CD107a and IL-2 expression were all observed to be significantly higher in individuals with HAM/TSP compared to AC. Interestingly, TNF-α expression was highest in SC and decreased after stimulation with HTLV-1 virus in all groups. These data indicate that both *ex vivo* baseline and *in vitro* stimulated CD8 T cells from HAM/TSP subjects express higher levels of intracellular cytokines despite expressing higher levels of NCRs.

### Select NCR Blockade Results in Greater Anti-HTLV-1 CD8 T-Cell Cytokine Responses in AC Compared to HAM/TSP Subjects

**Figure 7:**
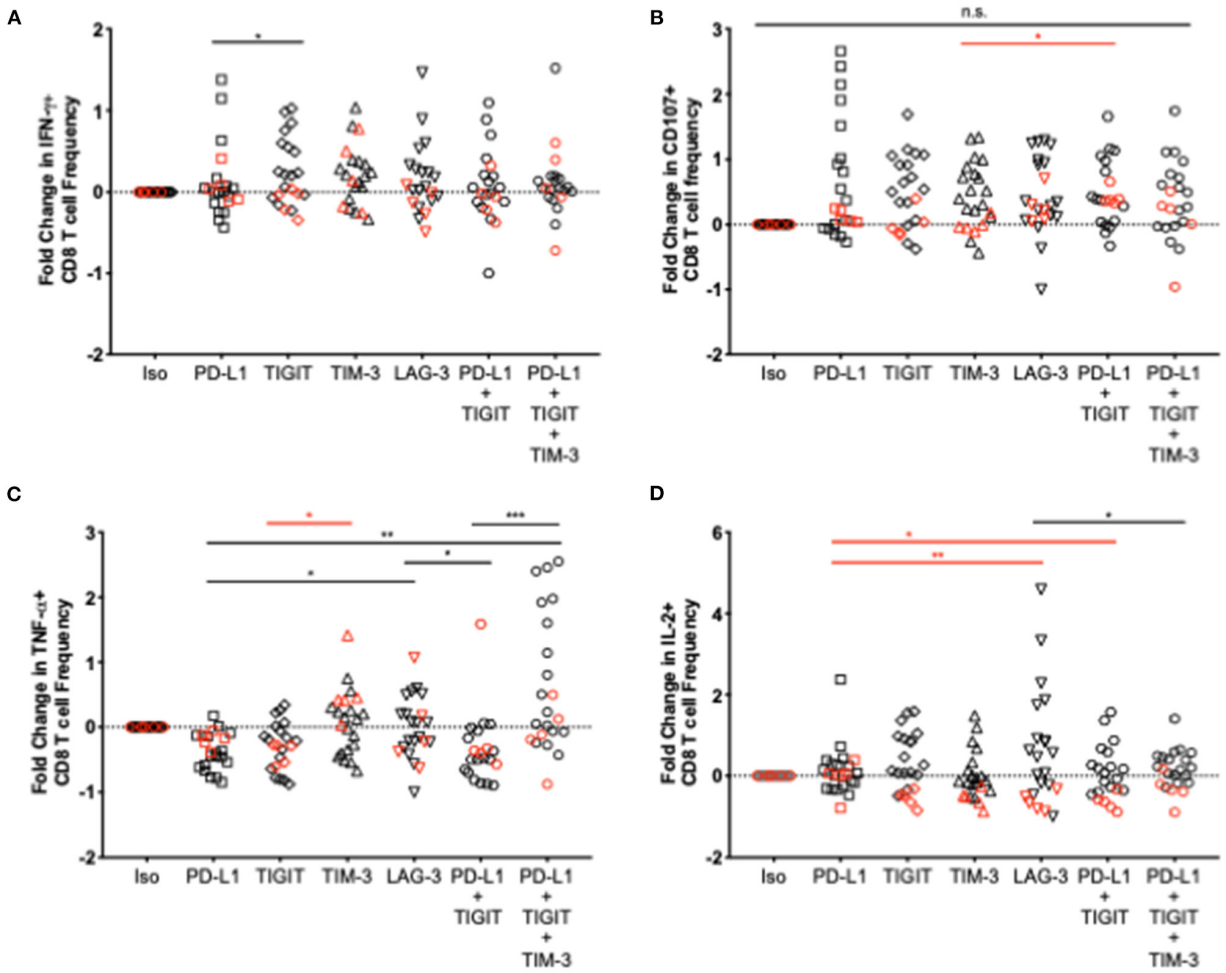
Effect of various single and combination blockade strategies on cytokine expression in CD8 T cells in HTLV-1 infection. Humanized IgG blocking antibodies against PD-L1, TIGIT, TIM-3, and LAG-3 or an appropriate isotype control were added individually or in combination to thawed PBMCs from HTLV-1- infected AC and individuals with HAM/TSP. PBMCs were then stimulated with whole virus concentrated from MT-2 cell culture supernatant. After a 16-h incubation and expansion of virus-specific CD8 T cells, cells were washed and stained with anti-IFN-γ **(A)**, anti-CD107a **(B)**, anti-TNF-α **(C)**, and anti-IL-2 **(D)** for assessment of changes in CD8 T-cell cytokine expression after various single and combination blockade strategies (x axis). Significant differences between blockades were calculated using the Friedman multiple comparisons test with *p*-values < 0.05 considered to be significant (*p < 0.05; **p < 0.005; ***p < 0.0005).

To assess the effect of various NCR blockade strategies on HTLV-1 specific CD8 T-cell function, clinical grade, fully humanized monoclonal antibodies targeting one or more NCRs were added to thawed PBMCs from HTLV-1-infected donors and were stimulated with HTLV-1 to capture virus-specific cytokine expression resulting from checkpoint blockade. Fold change (frequency of CD8 T cells after blockade – frequency of CD8 T cells in response to isotype control)/frequency of isotype control) was used as the measure of cytokine response. Both single and combination blockade strategies enhanced CD8 T-cell function to a greater degree in AC than in individuals with HAM/TSP (Figure 7). Significant differences in cytokine expression between single and combination blockade were observed for TNF-α and IL-2 in AC and HAM/TSP, respectively. Furthermore, triple combination blockade significantly increased TNF-α expression in CD8 T cells from AC to a greater degree than dual PD-L1/TIGIT blockade. Due to the limited number of cells available for blockade testing, we were unable to assess additional triple and quadruple NCR combinations. Since TIM-3 expression was found to be significantly upregulated in HAM/TSP subjects, this selected over LAG-3 for the triple combination blockade studies.

### NCR Blockade Decreases Anti-HTLV-1 IL-2 Expression in CD8 T Cells in HAM/TSP Subjects but Not AC

We observed a significantly decreased IL-2 expression following *in vitro* blockade in almost all individuals regardless of blockade strategy in the HAM/TSP group. Except for several individuals in the single PD-L1 and triple blockade groups, both single and combination blockade strategies resulted in a markedly significant decrease in IL-2^+^ CD8 T cell responses. TIGIT, TIM-3, LAG-3, and dual TIGIT/PD-1 blockade each yielded a consistent reduction in IL-2^+^ CD8 T cell responses across all individuals in the HAM/TSP group. Decreased IL-2^+^ CD8 T cell responses in the AC group were also observed in select donors', but not to the same extent, and not as consistently across all individuals for each blockade.

## Discussion

It is currently unclear which viral, host and immune components are responsible for driving HTLV-1-associated diseases, and why only some individuals become symptomatic. Numerous studies have reported an association between T-cell exhaustion and NCR expression (3, 5, 7, 24), and in addition to exuberant T cell activity in HTLV-1-infected individuals (25, 26), impaired T-cell activity may be an alternative hypothesized mechanism contributing to disease pathogenesis (6, 7, 27) An expanded profile of 2nd and 3rd tier NCR pathways continue to be discovered and evaluated. The objective of this study was to assess the expression of multiple NCRs on CD8 T cells in HAM/TSP subjects and determine whether combination immune checkpoint blockade strategies effectively enhance anti-HTLV-CD8 T-cell function.

HAM/TSP patients, who suffer from debilitating neurological and motor dysfunction, showed increased TIM-3 and LAG-3 expression on CD4 and increased TIM-3 expression CD8 T cells, indicating that these two NCRs may be important regulators of T-cell function in HTLV-1 infection. Unsurprisingly, PD-1 expression was not elevated, confirming the results of previous studies that assessed PD-1 expression in HAM/TSP subjects (28). More importantly, significant increases in triple and quadruple NCR expression on both CD4 and CD8 T cells from HAM/TSP subjects suggest that co-expression of multiple NCRs may be an important driver of HTLV-1 associated neurological disease. Previous studies have also shown hierarchical loss of T-cell function with increased expression of multiple NCRs. Still, single NCR expression patterns may point to hierarchical significance of certain NCRs in driving HTLV-1 pathogenesis (29). When assessing NCR expression on specific T-cell subsets, we observed increases in triple and quadruple NCR expression, particularly on CM CD8 T cells. Since this particular T-cell subset is important for homing to secondary lymphoid tissues, increased expression of multiple NCRs on these important T-cell subsets may indicate a mechanism by which HTLV-1 promotes immune evasion of anti-HTLV-1 immunity in order to ensure viral persistence and disease progression.

Evidence also suggests that high proviral load and constant expression of the viral transactivator protein Tax promotes HTLV-1 disease development and progression (30–34). As expected, in our study proviral load was higher in HAM/TSP subjects but did not correlate with higher frequencies of HTLV-1 Tax-specific CD8 T cells in asymptomatic carriers or HAM/TSP subjects. NCR expression on Tax-specific CD8 T cells did not differ significantly between AC and HAM/TSP subjects. A larger spread in the frequency of Tax-specific CD8 T cells was observed in AC, indicating that the number of virus-specific CD8 T cells among infected individuals varies more substantially in asymptomatic infection. Our ability to detect Tax-specific CD8 T cells was limited by the single epitope Tax pentamer used (Tax 11-19) and HLA-A02-restricted binding assessed given the limited repertoire of HLA-restricted Tax specific epitopes available that can be mapped. Thus, although NCR expression on Tax-specific CD8 T cells did not reveal significant differences between AC and HAM/TSP subjects, it is possible that increased sample size or pentamer availability for more HLA types and epitopes could reveal intergroup differences.

Given that CD8^+^ T cells in HTLV-1 infection may paradoxically contribute to the immunopathology and the clinical manifestation of HAM/TSP through an inflammatory state (25, 26), it is conceivable that the timing and the dynamics of events of inflammation vs. immune exhaustion co-exist in the evolution of immune failure in controlling infection and exacerbated pathology. This is noticeable in the setting of HIV where an immune deficiency is associated with persistent inflammation that has been linked to long-term organ specific complications (23, 35). Assessment of immune exhaustion and measures of inflammation over time in longitudinal studies would be informative to resolve this paradox.

It is also important to consider the expression of NCR ligands on APCs, as they are important for negative immune checkpoints. Both PD-L1 and PVR were found to be elevated in HAM/TSP subjects. Interestingly, the elevated expression of PD-L1 on mDCs and pDCs in HAM/TSP did not mirror that of PD-1 on CD4 and CD8 T cells, suggesting that increases in NCR ligand expression may not drive increased expression of cognate receptors. However, increased ligand availability may still be relevant in modulating T-cell function.

Previous studies have shown that inhibitory receptor signaling dampens effector T-cell responses, while decreasing T-cell proliferation, though HTLV-1 can circumvent this. HBZ, a viral oncoprotein expressed by the HTLV-1 transcript's complement strand, has been shown to induce NCR expression while also blocking suppression of infected cell proliferation (36). Consequently, HTLV-1-infected cells proliferate *in vivo* despite upregulated NCR expression. CD151/Ki67 co-expression represents a hyperproliferative phenotype of T cells, which was shown to be increased on HAM/TSP CD4 T cells. This supports our general understanding of HTLV-1 pathogenesis: expansion of infected CD4 T-cell clones eventually outpaces the immune response capacity to clear infected cells, though markers of replicative senescence on CD4 T cells in individuals with HAM/TSP do not differ from AC.

Key CD8 T-cell cytokines important in antiviral immunity (37, 38) are expressed at significantly higher levels in HAM/TSP subjects than in AC, indicating CD8 T-cell function is not severely compromised in HAM/TSP despite elevated expression of exhaustion markers. Interestingly, T-cell exhaustion has been shown to exist on a spectrum, with level of exhaustion depending on several factors including CD4 T-cell help and viral antigen levels (4). CD8 T-cell expression of various cytokines, particularly IL-2, TNF-α and IFN-γ, indicates the degree of exhaustion. Nonetheless, it is still possible for exhausted T cells to produce cytokines. Decreases in CD4 T-cell help and increases in antigen levels during viral infection result in increased NCR expression and a hierarchical loss of cytokine production as exhaustion becomes terminal. Loss of IL-2 is an early indicator of exhaustion, followed by loss of TNF-α and finally IFN-γ in severely exhausted states (27, 39, 40). Our data suggest that HTLV-1 infection may not follow the same pattern of exhaustive markers seen in other chronic viral infections (like lymphocytic choriomeningitis) (39), since IL-2 levels remain elevated in individuals with HAM/TSP. TNF-α expression, however, did seem to decrease relative to seronegative controls. IFN-γ expression was high in two HAM/TSP subjects and low in others, which suggests that exhaustion is indeed a spectrum, and that some individuals may be experiencing more T-cell exhaustion than others. Interestingly, IFN-γ expression in HAM/TSP subjects decreases after antigenic stimulation, with minute increases observed for CD107a and TNF-α. HTLV-1-specific CD8 T cells have been shown to lack recall responses to HTLV-1 antigen, which may explain the decrease in IFN-γ expression upon stimulation (28). The reintroduction of HTLV-1 antigen could also be driving further states of T-cell exhaustion, hence the decrease in IFN-γ expression.

A recent study suggests that HTLV-1-intrinsic factors may regulate TIGIT expression. TIGIT on CD4 T cells increased in individuals with ATL and the frequency of IFN-γ-secreting cells increased in some individuals following TIGIT blockade (10). Moreover, dual anti-TIGIT and anti-PD-1 blockade resulted in further increases in IFN-γ secreting cells in addition to higher proportions of responding individuals, highlighting the potential added benefit of blocking multiple NCRs to enhance CTL function. Increases in IFN-γ expression were observed in only one HAM/TSP subject in this study upon both PD-1 and TIGIT blockade. However, dual blockade resulted in greater CD107a expression in almost all HAM/TSP subjects. Triple blockade resulted in consistently higher levels of IFN-γ, CD107a, and TNF-α expression, suggesting that blocking multiple NCRs has synergistic effects. This effect was also seen in AC, particularly regarding TNF-α expression. The addition of TIM-3 blockade to dual PD-1 and TIGIT blockade resulted in increased IFN-γ and TNF-α expression in HAM/TSP subjects, which may indicate hierarchical importance of TIM-3 in HTLV-1 infection. These findings support the hierarchical importance of NCRs and that devising strategies to block these receptors in order to boost T-cell function likely depends on the specific CTL response that is desired.

When comparing AC and HAM/TSP following blockade, the degree of cytokine expression between the two groups was noticeably different, suggesting that carrier status vs. disease states may also be important to consider when devising blockade strategies. Certain strategies may be useful for preventing disease development, and others for curbing disease progression. The decrease in IL-2 expression observed in the HAM/TSP group after immune check blockade is intriguing. While IL-2 can be used as a measure of effective T-cell function and is produced in large quantities upon antigen-mediated activation, it is part of a feedback loop that promotes the maintenance of regulatory T cells. Checkpoint blockade in HAM/TSP subjects may limit IL-2 production in CD cells to control the proliferation of infected CD4 T cells. Measuring Ki67 expression in CD4 T cells before and after checkpoint blockade would elucidate whether this IL-2 reduction plays a direct role in limiting the clonal expansion of virus-infected cells.

The influence of T cell dysfunction, following increased NCR expression, on HTLV-1 disease progression is not fully understood. However, the results generated in this study suggest that HAM/TSP subjects retain a reduced capacity of HTLV-1 specific CD8 T cells to respond to checkpoint blockade when compared to AC, likely due to differences in the degree of T-cell exhaustion. It is clear that different disease states may benefit from different blockade strategies and that combination strategies may be more effective for boosting certain T-cell responses. These results serve to better guide future *in vivo* blockade studies that may yield new treatments for HTLV-1-associated diseases.

## Data Availability Statement

The raw data supporting the conclusions of this article will be made available by the authors, without undue reservation.

## Ethics Statement

The studies involving human participants were reviewed and approved by University of Hawaii Human Studies Program. The patients/participants provided their written informed consent to participate in this study.

## Author Contributions

DC, GC, LN, and PJ: conceived and designed experiments. DC, GC, and ED: performed experiments. DC, GC, BC, ED, and RB: analyzed the data. EM, LN, and PJ: contributed reagents and materials. DC, BC, GC, ED, RB, EM, LN, and PJ: contributed to manuscript writing. All authors contributed to the article and approved the submitted version.

## Conflict of Interest

The authors declare that the research was conducted in the absence of any commercial or financial relationships that could be construed as a potential conflict of interest.
