# Supplementary material for: Phenotypic and Functional Analyses Guiding Combination Immune Checkpoint Immunotherapeutic Strategies in HTLV-1 Infection
Source: Front Immunol. 2021 Mar 9;12:608890. doi: 10.3389/fimmu.2021.608890 (PMC7985073; doi:10.3389/fimmu.2021.608890)
Supplement: Supplementary file 1 [file Data_Sheet_1.PDF]

Supplementary Material

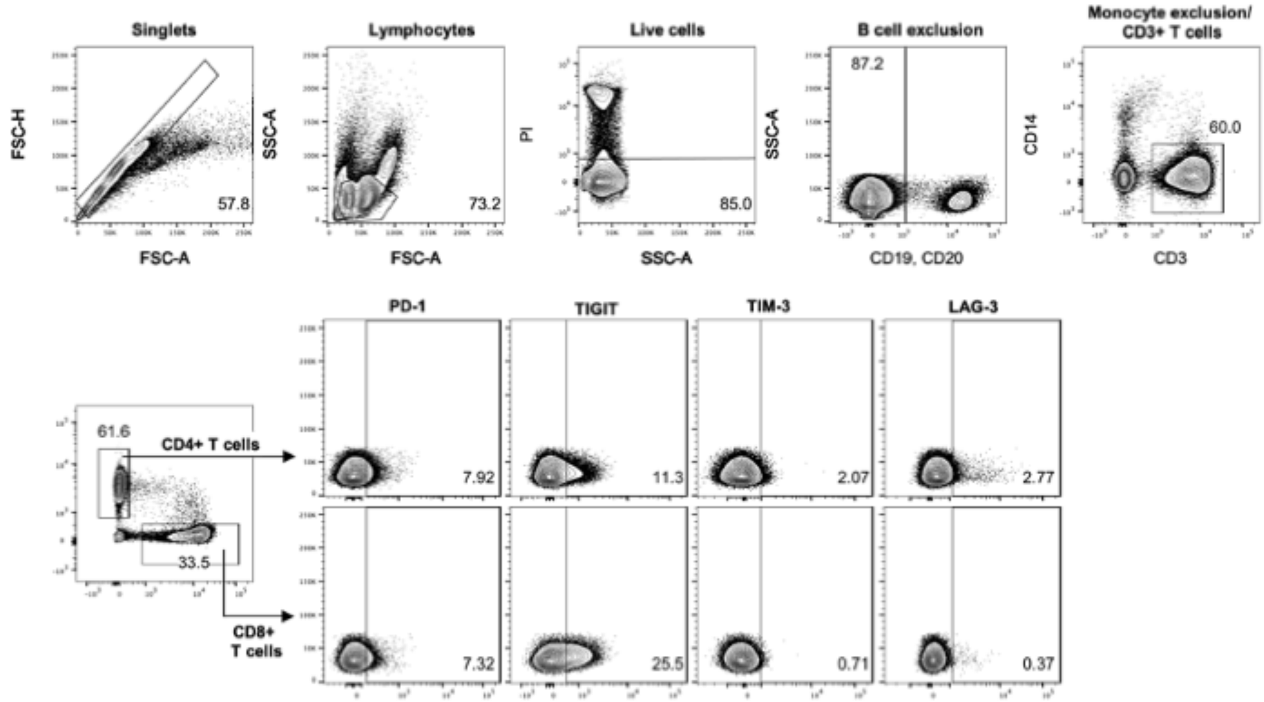

Supplementary Figure 1. Representative flow cytometry plots showing the gating strategy used to phenotype CD4 and CD8 T cells and assess NCR expression are shown. First, singlets were gated, followed by selection of lymphocytes based on size and granularity. Dead cells were excluded based on propidium iodide staining. Next, B cells were excluded based on CD19/CD20 expression. Finally, T cells were selected using a $CD3^{+}CD14^{-}$ gate (for monocyte exclusion). CD4 and CD8 T cells were then assessed for expression of PD-1, TIGIT, TIM-3 and LAG-3.

.

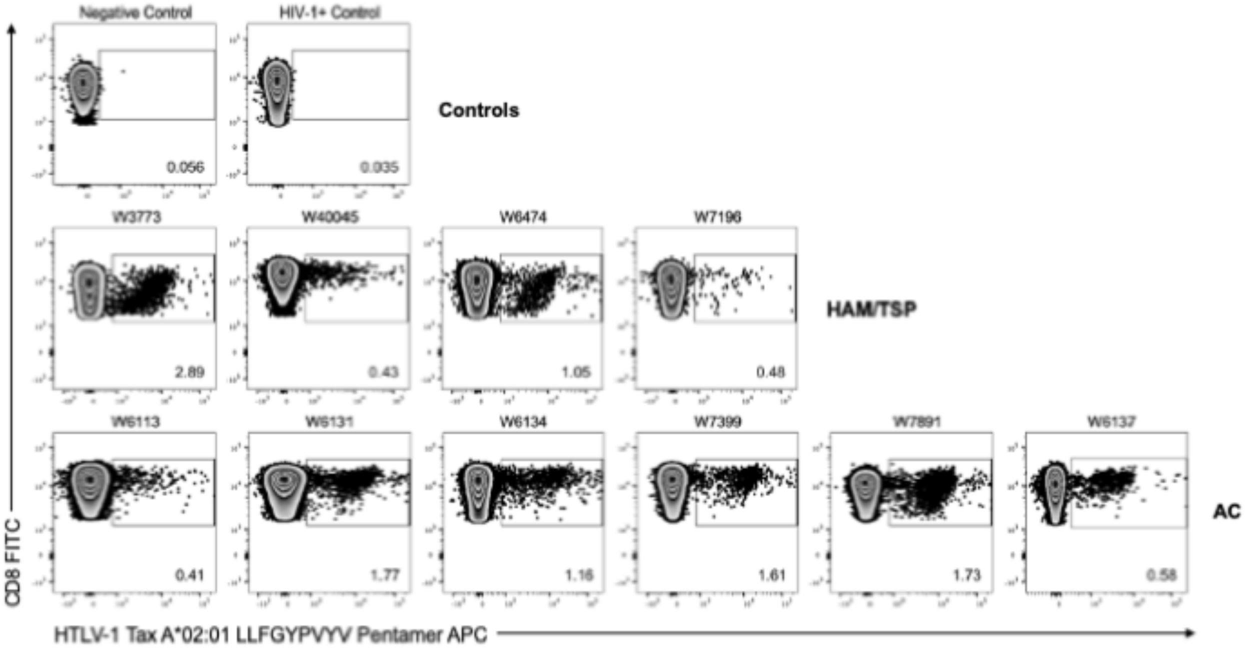

Supplementary Figure 2. A*02:01 Tax$_{11-19}$ pentamer staining of uninfected controls, individuals with HAM/TSP (n=4) and AC (n=6) are shown. Uninfected controls included an HTLV-1 uninfected and HIV+ sample and were used to set the pentamer positive gate. Non-specific staining was accounted for using a negative control pentamer. Pentamer staining is shown on gated CD8^{+} T cells.

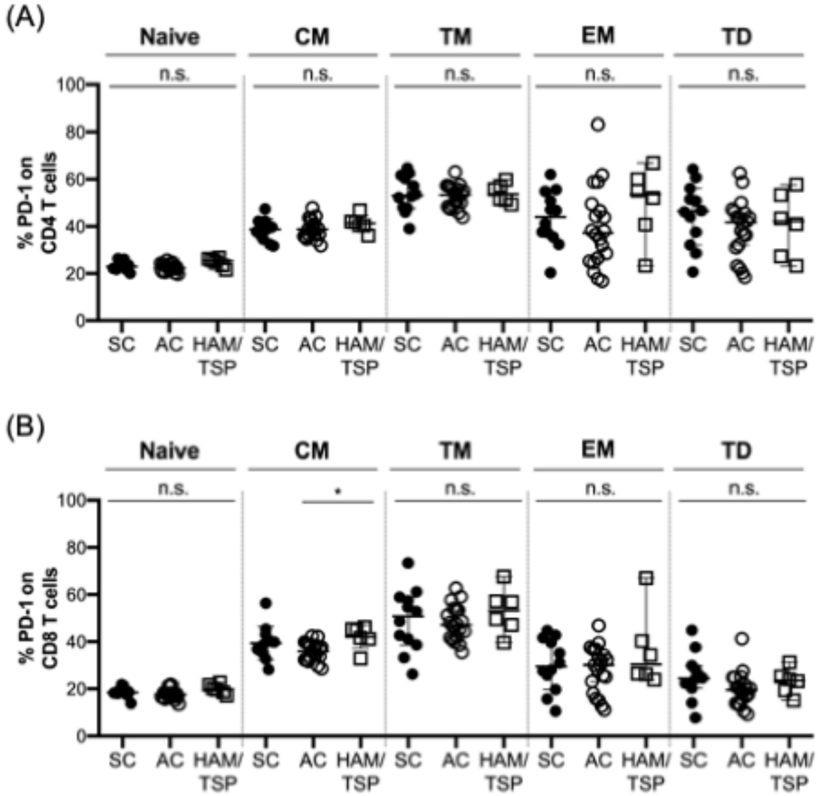

Supplementary Figure 3. Cryopreserved PBMCs were used to immunophenotype memory T-cell subsets and assess expression of PD-1 on (A) CD4 T cells and (B) CD8 T cells from seronegative controls (SC), HTLV-1- positive asymptomatic carriers (AC), and individuals with HAM/TSP (HAM/TSP). Memory T-cell subsets were determined by differential expression of CD28, CD45RA and CCR7 as follows: naïve ($CD28^{+}CD45RA^{+}CCR7^{+}$), central memory (CM, $CD28^{+}CD45RA^{-}CCR7^{+}$), transitional memory (TM, $CD28^{+}CD45RA^{-}CCR7^{-}$), effector memory (EM $CD28^{-}CD45RA^{-}CCR7^{-}$) and terminally differentiated (TD, $CD28^{-}CD45RA^{+}CCR7^{-}$). Significant differences between groups were calculated using Kruskal-Wallis multiple comparisons test with p-values < 0.05 considered to be significant (*p < 0.05; **p < 0.005; ***p < 0.0005).

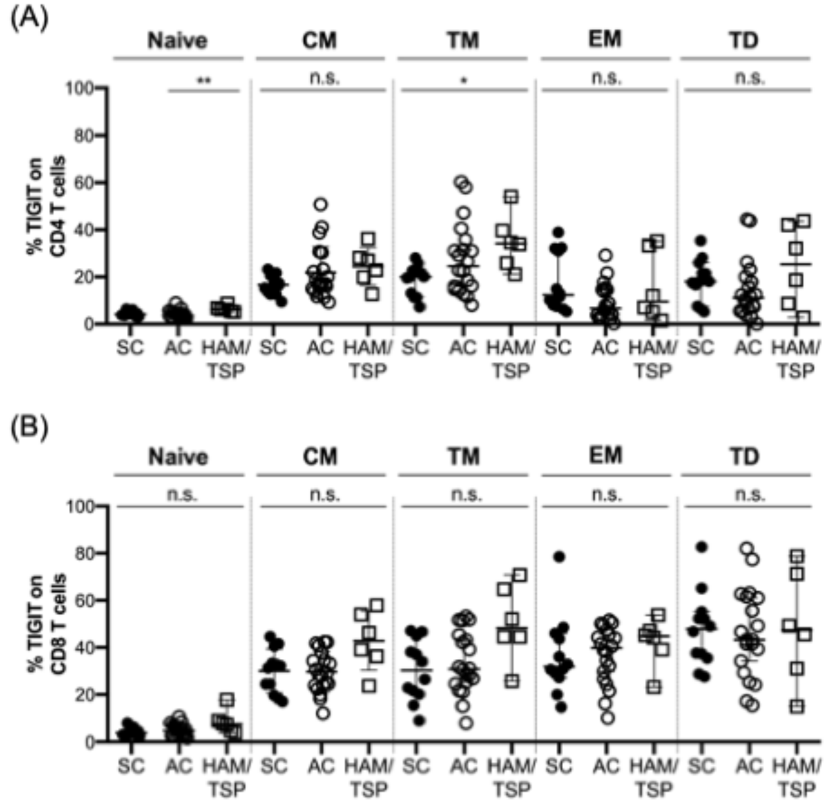

Supplementary Figure 4. Cryopreserved PBMCs were used to immunophenotype memory T-cell subsets and assess expression of TIGIT on (A) CD4 T cells and (B) CD8 T cells from seronegative controls (SC), HTLV-1- positive asymptomatic carriers (AC), and individuals with HAM/TSP (HAM/TSP). Memory T-cell subsets were determined by differential expression of CD28, CD45RA and CCR7 as follows: naïve ($CD28^{+}CD45RA^{+}CCR7^{+}$), central memory (CM, $CD28^{+}CD45RA^{-}CCR7^{+}$), transitional memory (TM, $CD28^{+}CD45RA^{-}CCR7^{-}$), effector memory (EM $CD28^{-}CD45RA^{-}CCR7^{-}$) and terminally differentiated (TD, $CD28^{-}CD45RA^{+}CCR7^{-}$). Significant differences between groups were calculated using Kruskal-Wallis multiple comparisons test with p-values < 0.05 considered to be significant (*p < 0.05; **p < 0.005; ***p < 0.0005).

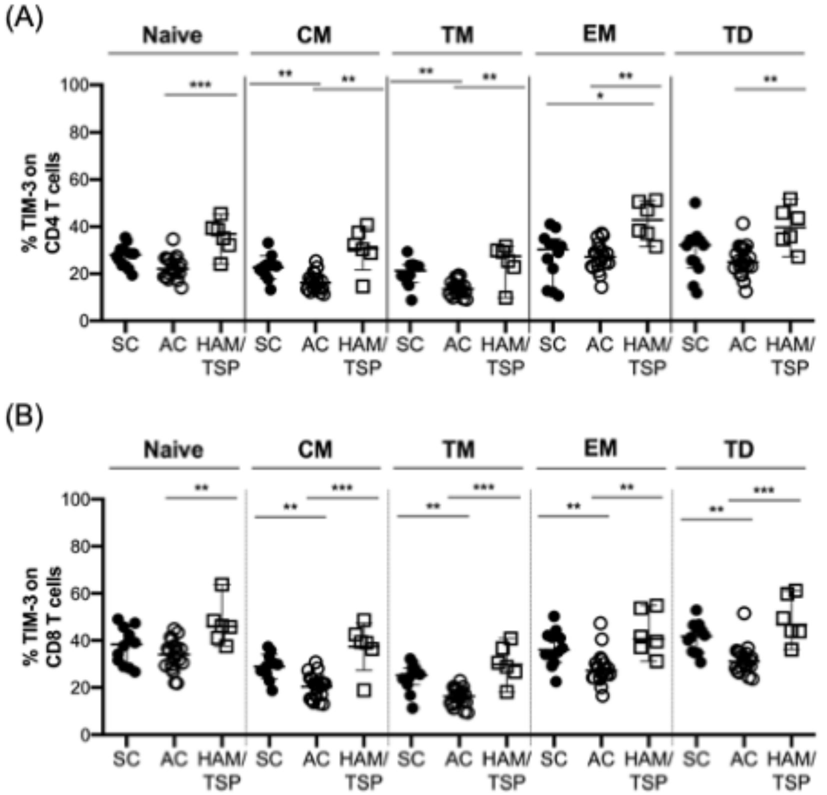

Supplementary Figure 5. Cryopreserved PBMCs were used to immunophenotype memory T-cell subsets and assess expression of TIM-3 on (A) CD4 T cells and (B) CD8 T cells from seronegative controls (SC), HTLV-1- positive asymptomatic carriers (AC), and individuals with HAM/TSP (HAM/TSP). Memory T-cell subsets were determined by differential expression of CD28, CD45RA and CCR7 as follows: naïve ($CD28^{+}CD45RA^{+}CCR7^{+}$), central memory (CM, $CD28^{+}CD45RA^{-}CCR7^{+}$), transitional memory (TM, $CD28^{+}CD45RA^{-}CCR7^{-}$), effector memory (EM $CD28^{-}CD45RA^{-}CCR7^{-}$) and terminally differentiated (TD, $CD28^{-}CD45RA^{+}CCR7^{-}$). Significant differences between groups were calculated using Kruskal-Wallis multiple comparisons test with p-values < 0.05 considered to be significant (*p < 0.05; **p < 0.005; ***p < 0.0005).

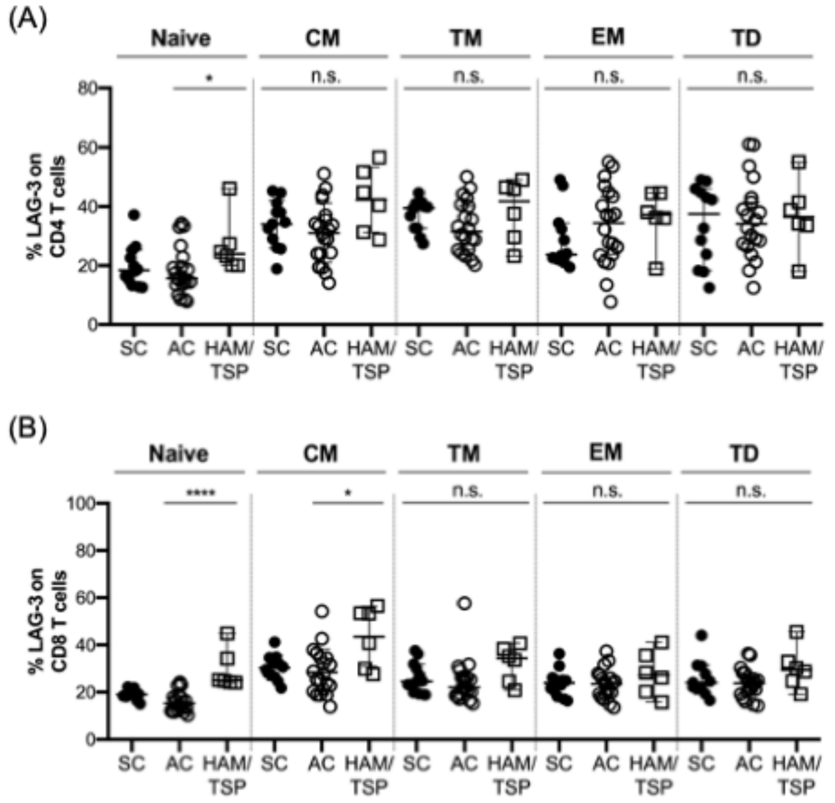

Supplementary Figure 6. Cryopreserved PBMCs were used to immunophenotype memory T-cell subsets and assess expression of LAG-3 on (A) CD4 T cells and (B) CD8 T cells from seronegative controls (SC), HTLV-1- positive asymptomatic carriers (AC), and individuals with HAM/TSP (HAM/TSP). Memory T-cell subsets were determined by differential expression of CD28, CD45RA and CCR7 as follows: naïve ($CD28^{+}CD45RA^{+}CCR7^{+}$), central memory (CM, $CD28^{+}CD45RA^{-}CCR7^{+}$), transitional memory (TM, $CD28^{+}CD45RA^{-}CCR7^{-}$), effector memory (EM $CD28^{-}CD45RA^{-}CCR7^{-}$) and terminally differentiated (TD, $CD28^{-}CD45RA^{+}CCR7^{-}$). Significant differences between groups were calculated using Kruskal-Wallis multiple comparisons test with p-values < 0.05 considered to be significant (*p < 0.05; **p < 0.005; ***p < 0.0005).

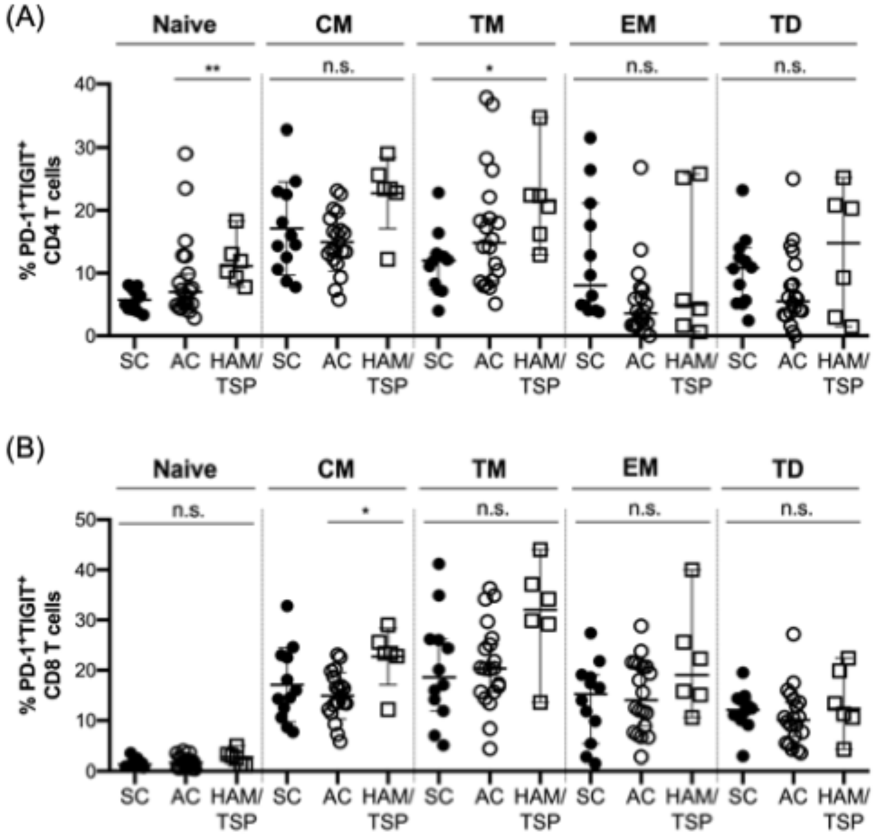

Supplementary Figure 7. Cryopreserved PBMCs were used to immunophenotype memory T-cell subsets and assess co-expression of PD-1 and TIGIT on (A) CD4 T cells and (B) CD8 T cells from seronegative controls (SC), HTLV-1- positive asymptomatic carriers (AC), and individuals with HAM/TSP (HAM/TSP). Memory T-cell subsets were determined by differential expression of CD28, CD45RA and CCR7 as follows: naïve ($CD28^{+}CD45RA^{+}CCR7^{+}$), central memory (CM, $CD28^{+}CD45RA^{-}CCR7^{+}$), transitional memory (TM, $CD28^{+}CD45RA^{-}CCR7^{-}$), effector memory (EM $CD28^{-}CD45RA^{-}CCR7^{-}$) and terminally differentiated (TD, $CD28^{-}CD45RA^{+}CCR7^{-}$). Significant differences between groups were calculated using Kruskal-Wallis multiple comparisons test with p-values < 0.05 considered to be significant (*p < 0.05; **p < 0.005; ***p < 0.0005).

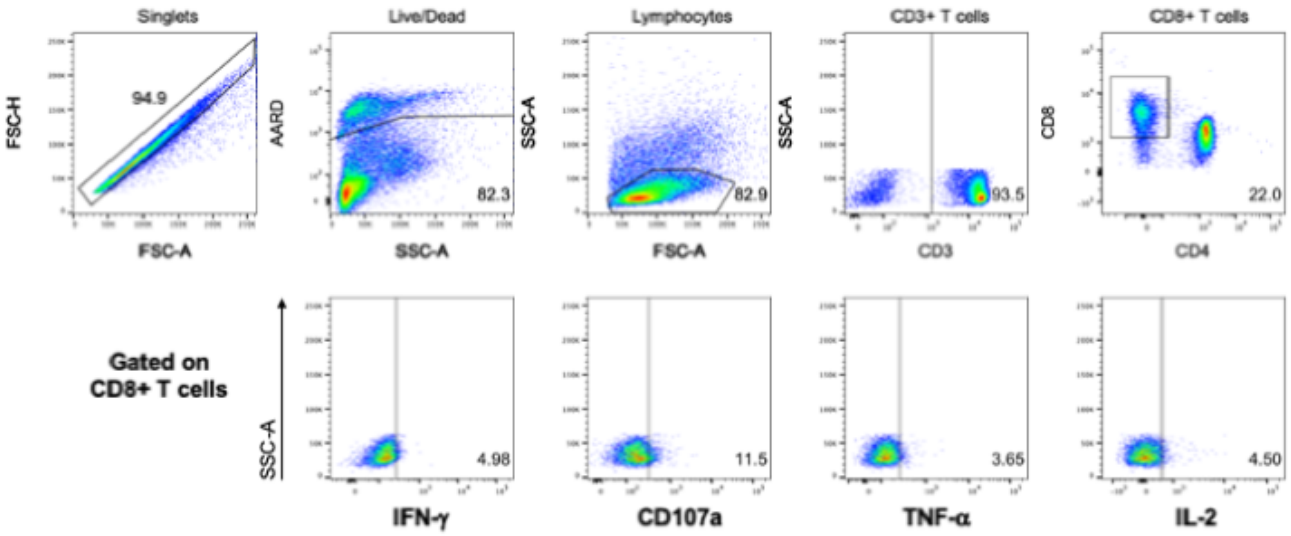

Supplementary Figure 8. Representative flow cytometry gating for cytokine expression in $CD8^{+}$ T cells are shown. PBMCs stimulated with HTLV-1 whole virus and blocked with various single and combination blocking antibodies were stained for viability (AARD), T cell markers (CD3, CD4 and CD8) and intracellular cytokines (IFN-γ, CD107a, TNF-α and IL-2). First, doublets were excluded followed by exclusion of dead cells. Lymphocytes were gated based on size and granularity. Intracellular cytokine expression was then assessed on $CD3^{+}CD8^{+}$ T cells. FMOs were used to approximate positive gates
